# A comparison of random forest variable selection methods for regression modeling of continuous outcomes

**DOI:** 10.1093/bib/bbaf096

**Published:** 2025-03-10

**Authors:** Nathaniel S O’Connell, Byron C Jaeger, Garrett S Bullock, Jaime Lynn Speiser

**Affiliations:** Department of Biostatistics and Data Science, Wake Forest University School of Medicine, Medical Center Boulevard, Winston-Salem, NC 27157, United States; Department of Biostatistics and Data Science, Wake Forest University School of Medicine, Medical Center Boulevard, Winston-Salem, NC 27157, United States; Department of Biostatistics and Data Science, Wake Forest University School of Medicine, Medical Center Boulevard, Winston-Salem, NC 27157, United States; Department of Orthopedic Surgery, Wake Forest University School of Medicine, Medical Center Boulevard, Winston-Salem, NC 27157, United States; Department of Biostatistics and Data Science, Wake Forest University School of Medicine, Medical Center Boulevard, Winston-Salem, NC 27157, United States

**Keywords:** random forest, variable selection, feature selection, feature reduction, regression

## Abstract

Random forest (RF) regression is popular machine learning method to develop prediction models for continuous outcomes. Variable selection, also known as feature selection or reduction, involves selecting a subset of predictor variables for modeling. Potential benefits of variable selection are methodologic (i.e. improving prediction accuracy and computational efficiency) and practical (i.e. reducing the burden of data collection and improving efficiency). Several variable selection methods leveraging RFs have been proposed, but there is limited evidence to guide decisions on which methods may be preferable for different types of datasets with continuous outcomes. Using 59 publicly available datasets in a benchmarking study, we evaluated the implementation of 13 RF variable selection methods. Performance of variable selection was measured via out-of-sample *R*^2^ of a RF that used the variables selected for each method. Simplicity of variable selection was measured via the percent reduction in the number of variables selected out of the number of variables available. Efficiency was measured via computational time required to complete the variable selection. Based on our benchmarking study, variable selection methods implemented in the *Boruta* and *aorsf* R packages selected the best subset of variables for axis-based RF models, whereas methods implemented in the *aorsf* R package selected the best subset of variables for oblique RF models. A significant contribution of this study is the ability to assess different variable selection methods in the setting of RF regression for continuous outcomes to identify preferable methods using an open science approach.

## Introduction

Random forest (RF) regression is a frequently used machine learning algorithm to develop models for continuous outcomes. First introduced in 2001 [1], RFs are a collection of classification and regression decision trees [2] which are simple models using binary splits on predictor variables that are used to ascertain predictions. In a regression setting, the RF computes predictions by taking the mean of predictions from each decision tree within the forest. Decision trees are easy to use in practice, offering an intuitive method for modeling by splitting variables into subgroups; however, their simplicity often results in poor accuracy [1]. Prior studies have shown RFs can provide better prediction accuracy compared to a single decision tree, regression model, and other machine learning algorithms [3]. RFs allow for interpretation of relationships between predictors and outcome using variable importance measures [4].

In addition to developing prediction models, RF can also be used to conduct variable selection, a process in which a subset of predictors is selected for inclusion a model. Also known as feature selection or feature reduction, variable selection can improve prediction accuracy and decrease the burden of applying prediction models in practice. For example, rather than using all variables available in a registry dataset, one may prefer to use only a subset of the most important variables when developing a model [5, 6]. In prediction modeling, an interest is often to determine a minimal set of the most important predictors that should be included in a reduced, parsimonious model. This can be achieved by performing variable selection, in which optimal predictors are identified based on characteristics such as importance or accuracy [6]. Developing prediction models using variable selection may reduce the burden of data collection required to make predictions for new observations, thereby improving the efficiency of prediction and uptake of model use in practice. For datasets with many possible predictor variables, variable selection is a critical step as it can remove superfluous variables that are unnecessary for modeling and improve the prediction accuracy of downstream models (i.e. models that are trained using the selected subset of variables). Therefore, many benefits of variable selection exist, and appropriate variable selection should be considered in bioinformatics analyses.

There are several methods available for performing variable selection in the setting of RF regression for continuous outcome modeling using a variety of approaches (test based and performance based) and RF implementations (axis based, conditional inference, and oblique). While there are many methods for RF variable selection for regression problems, the current literature lacks general comparisons between existing methods using real data that can offer empirical guidance about which methods are preferable in terms of model performance, simplicity, and computational efficiency. Sanchez-Pinto [7], Degenhardt [8], Cadenas [9], Hapfelmeier [10], and Speiser [11] assessed variable selection methods for classification of categorical outcomes, but less studies have evaluated RF variable selection methods for regression of continuous outcomes [8, 12]. RF variable selection methods may have differential performance in classification versus regression due to differing outcome definitions, performance statistics, and criteria for growing decision trees. For categorical outcomes, metrics such as prediction accuracy (percent of correct predictions) may be used to add or eliminate variables, whereas for continuous outcomes, root mean square error is used. It is unclear if performance metrics may behave in different ways within the variable selection methods for continuous outcomes [13]. To provide data-driven recommendations for regression, we aimed to conduct a benchmarking study using publicly available data to assess implementations of RF variable selection methods [14].

The remainder of this paper is structured in the following manner. We first summarize methods and implementations for RF variable selection for regression in the current literature. Next, the design of the current study is presented, including the datasets used and evaluation metrics for the variable selection procedures. We then provide a summary of results comparing performance, parsimony, and computation time for the implementations of the variable selection methods. Finally, discussion and conclusions are presented.

## Methods for random forest variable selection for regression

Our goal was to be as inclusive as possible in terms of using all available variable selection methods for RF regression with available R code to thoroughly evaluate and compare methods with specific implementations. Several R packages are available for implementing RF variable selection, including *caret* [15], *vita* [16], *boruta* [17], *RRF* [18], *randomForestSRC* [19], *VSURF* [20], and *rfvimptest* [10, 12]. Methods proposed by Svetnik [21] and Jiang [22] utilize the R package *party* [23]. The methods by Menze [24] and Jaeger (method referred to as Negation) [25] based on oblique RFs are implemented within *aorsf* [26]. The *aorsf* package offers three approaches, each of which conducts recursive feature elimination: aorsf-Menze, aorsf-Negation, and aorsf-Permutation. Lastly, a few methods are only available for classification problems and do not offer a regression alternative, including Janitza’s method [16], *varSelRF* [27] and *AUCRF* [28], and are therefore omitted from the analysis in this paper.

We included a total of 13 RF variable selection methods in this benchmarking study. Each method is described in detail by its corresponding paper, which is presented alongside a brief description of the method in Table 1. In brief, methods proposed by Svetnik, Jiang, Menze, methodology included in the R package *caret*, aorsf-Negation, and aorsf-Permutation each use recursive feature elimination, a method that recursively fits a RF and then drops the least important predictor, and then assesses out-of-bag prediction accuracy to identify an optimal set of predictors. Altmann’s method and the methods included in the R packages *Boruta* and *rfvimptest* use permutation tests to identify significant predictors. The method in the *RRF* R package uses forward selection based on regularization. The method in the *randomForestSRC* R package, which we denote SRC, uses minimal depth of maximal subtrees. The method in the *VSURF* R package employs a three-step selection procedure involving thresholding, interpreting, and predicting outcomes. We also included a minimally complex approach, Axis-SFE, which employs single-feature elimination in axis-based RFs, where permutation variable importance is computed, and all features with importance values greater than 0 are retained. The role of Axis-SFE in our analysis is to allow us to evaluate whether a simple and efficient variable selection approach performs as well as techniques that are more computationally expensive. Hyperparameters for the variable selection method implementations were based on the random forest type and therefore may differ for different methods. We used default parameter settings for most models, with a few exceptions that are listed in Table 1.

**Table 1:** Summary of variable selection methods for random forest regression

Abbreviation in paper	Publication	R package/implementation	Approach	Type of forest method	Summary	Parameter settings
None	Breiman 2001 [1]	*ranger*	N/A	Axis	No variable selection	Default
Svetnik	Svetnik 2004 [16]	Uses *party*, code from Hapfelmeier [15]	Performance based	Conditional inference	Uses recursive feature elimination^a^ based on importance measures and *k*-fold validation	# trees = 100, # folds = 5, # repetitions = 20
Jiang	Jiang 2004 [17]	Uses *party*, code from Hapfelmeier [15]	Performance based	Conditional inference	Similar to Svetnik but provides mechanism to prevent overfitting	# trees = 500, # standard error rule = minimum
Caret	Kuhn 2008 [8]	*caret*	Performance based	Axis	Uses recursive feature elimination,^a^ criteria to remove variables based on maintaining similar error rate to full model	Default
Altmann	Altmann 2010 [18]	*vita*	Test based	Axis	Based on a parametric test of repeated permutations of importance measures	Default
Boruta	Kursa 2010 [5]	*Boruta*	Test based	Axis	Based on a permutation test using a hold-out approach for importance measures	Default
aorsf-Menze	Menze 2011	*aorsf*	Performance based	Oblique	*P*-values are calculated for predictors at non-leaf nodes. Variable importance is based on the proportion of time a *P*-value for a predictor is <0.01. Recursive feature elimination is applied based on this importance metric^a^	predictor_min = 2
RRF	Deng 2013 [11]	*RRF*	Performance based	Axis	Based on a regularized random forest, which uses forward selection to sequentially add variables until there is no further information gain	Default
SRC	Ishwaran 2014 [10]	*randomForestSRC*	Performance based	Axis	Calculates the minimal depth of the maximal subtree (i.e. the largest subtree whose root node splits on the predictor) for each predictor	Default
VSURF	Genuer 2015 [7]	*VSURF*	Performance based	Axis	Three-step variable selection procedure. The “thresholding step” removes irrelevant variables. The “interpretation step” aims to select all variables related to the response for interpretation purpose. The “prediction step” refines the selection by eliminating redundancy in the set of variables selected by the second step	Default
aorsf-Negation	Jaeger 2023	*aorsf*	Performance based	Oblique	Variable importance is based on prediction accuracy after negating the signs of all coefficients linked to a variable. Recursive feature elimination is applied based on this importance metric^a^	Default
aorsf-Permutation	Jaeger 2023	*aorsf*	Performance based	Oblique	Variable importance is based on prediction accuracy after permuting values for a given variable. Recursive feature elimination is applied based on this importance metric^a^	Default
Axis-SFE		*ranger*	Test based	Axis	Selects all variables with variable importance >0 based on permutation	Default
rfvimptest	Hapfelmeier 2023 [15]	*rfvimptest*	Test based	Axis	Similar to Altmann, but uses unbiased importance measures	# type = SAPT

Methods are presented in chronological order by year of publication.

^a^Recursive feature elimination is a stepwise procedure where a single predictor is dropped at each step until a stopping criterion is met. At each step, out-of-bag prediction accuracy is computed. The set of variables that maximize out-of-bag prediction accuracy is determined as the final selection.

Similar to Hapfelmeier’s categorization [10] and our previous study [11], we define variable selection methods as being test based or performance based. Performance-based approaches select variables based on changes in the prediction accuracy of the continuous outcome when variables are added or deleted from models, and include methods by Svetnik, Jiang, and Menze, and methods in the R packages *caret*, *RRF*, *randomForestSRC*, and *VSURF*. Test-based approaches select variables based on statistical or permutation tests on the variables themselves, and include the method by Altmann and methods in the R packages *Boruta*, *aorsf* (aorsf-Menze, aorsf-Negation, aorsf-Permutation), *ranger* (Axis-SFE), and *rfvimptest*.

## Design of study

All datasets included in our benchmarking study are freely available with the *OpenML* (website: https://www.openml.org/) [29] and *modeldata* R packages [30]. We included datasets from these repositories designated as supervised regression tasks with <50% missing observations. We included datasets with 10–1000 predictors and 100–10,000 observations. Simulated datasets were excluded and redundant datasets (i.e. datasets in which there exist multiple versions) were limited to the most recent version. We required the continuous outcome to have at least 10 unique values.

**Figure 1:**
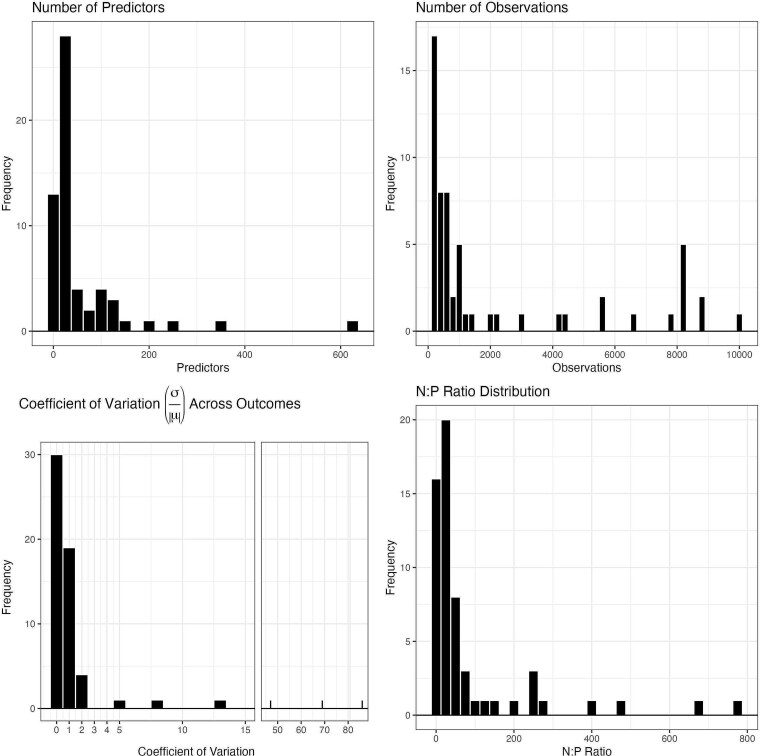
Characteristics of datasets used for the study. Distribution of number of predictors, total observations, coefficient of variation, and the N:P ratio for the 59 datasets used in the benchmarking study.

Based on these criteria, we analyzed a total of 59 data sets, 53 from the *OpenML* R package and 6 from the *modeldata* R package. We provide a summary of dataset characteristics in Fig. 1, which demonstrates the distributions for the number of predictors, number of observations, and coefficient of variation for the continuous outcome variable (CV; defined as standard deviation divided by the mean). In general, each of these characteristics was right skewed. The number of predictors ranged from 10 to 614, with a median of 22. The number of observations ranged from 120 to 10 000 with a median of 534. Most datasets (46; 78%) had a CV <1 indicating low variability relative to the mean, while 4 datasets had high variability with CV >10. The CVs ranged near 0 to 86, with a median of 0.48. The ratio of sample size to number of predictors (N:P) ranged from 1.54 to 769, with a median of 25.3. In total, 13 datasets had <10 observations per predictor (i.e. N:P ratio < 10). There were seven datasets that contained missing data in the predictors (11%), with missing proportions of 1%, 2%, 3%, 11%, 13%, 42%, and 50%. Details about the characteristics of datasets used in this study are included in Supplementary File 1. Datasets are from a variety of domains including medicine, manufacturing, weather, economics, education, and more.

We used 20 replications of split-sample validation (i.e. Monte Carlo cross-validation) for each dataset to evaluate implementations of the RF variable selection methods. First, a dataset was split into training (50%) and testing (50%) sets, except for datasets that had >2000 observations, for which we used 1000 randomly selected observations for the training data and the rest as testing data. Second, each variable selection method was applied to the training data, and the variables selected by each method were saved. For datasets with >150 predictors, at each replication, we selected a random subset of 150 variables to include in the variable selection method because computation time for these large datasets can be cumbersome. Third, each set of variables were used to fit an axis-based and oblique RF. Fourth, predictions for the testing data were computed with each RF. If any missing values were present in the training or testing data, they were imputed prior to running variable selection methods using the mean and mode for continuous and categorical predictors, respectively, computed in the training data.

For methods from available R packages, we used default parameters for each method, unless noted otherwise in Table 1. Computation time and the number of variables were recorded. We then fit a standard axis-based RF model using the R package *ranger* and an oblique RF using the package *aorsf* on the training dataset using variables selected from each method, and recorded *R*^2^ for each method from the test data. The *R*^2^ is a common statistic used to quantify overall prediction accuracy for continuous outcomes. It represents the percent of variation explained in an outcome (i.e. dependent variable) by the included predictors (i.e. independent variables) in the model, and ranges from 0 to 1 (or 0 to 100 if expressed as a percentage), where higher values represent better fit. For comparison, we fit a standard RF model using no form of variable selection. Some of the simulation runs for certain datasets and data splits resulted in some methods not selecting any variables. We recorded cases for which no variables were selected and forced the model to use the most important variable using the permutation importance metric as the one predictor variable included. We also provide a sensitivity analysis in which any simulation run containing a method that selected no variables was deleted for that dataset and replication for all methods.

We summarize results for computation time (and log-computation time) in seconds, percent variable reduction (i.e. the number of variables selected divided by the total number of variables available), and *R*^2^ aggregated across all dataset replications for each method. Additionally, we compare variable selection methods based on standardized metrics, in which for each dataset replication, we calculate *z*-scores for log-computation time, percent variable reduction, and *R*^2^ by variable selection method by dividing the mean of the metric by the standard deviation of the metric. These standardized outcomes allow us to compare how each method performs relative to other methods for a given replicate (i.e. random split) of a given dataset.

We used R version 4.3.0 on a computer with an AMD Ryzen 93900x 12-core/24-thread 3.8 GHz CPU with 2 × 16GB 3200 MHz DDR4 RAM. To ensure scientific rigor, authors N.S.O. and B.C.J. coded the benchmark study and author J.L.S. verified the code in an independent review. Code used to implement and evaluate the variable selection methods is provided on GitHub (https://github.com/NateOConnellPhD/rfvs_regression).

## Results

### Results for all datasets

**Figure 2:**
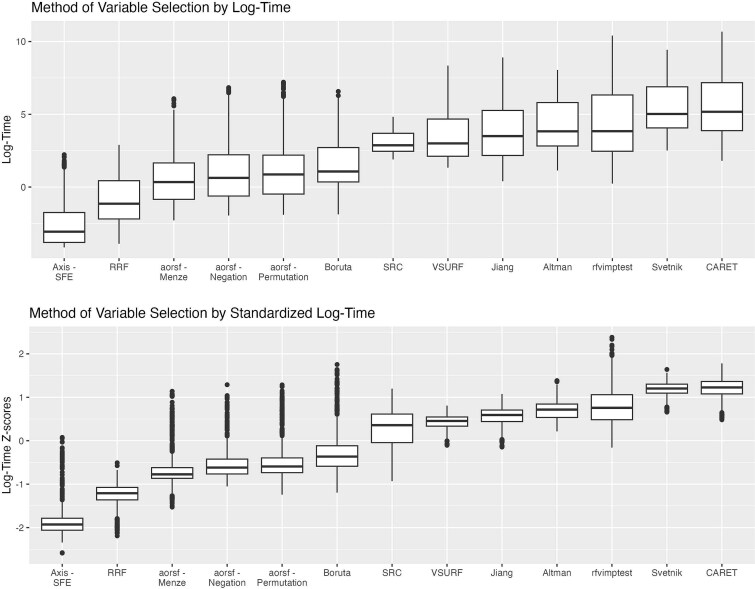
Distributions of log computation time and standardized log-computation time by method of variable selection. This shows the distribution of log-computation time and standardized log-computation time (*z*-scores) across all replication (20 replications per dataset) over each of the 59 datasets, by method of variable selection. The figure is ordered from left to right by median value in order of fastest to slowest computation time.

**Figure 3:**
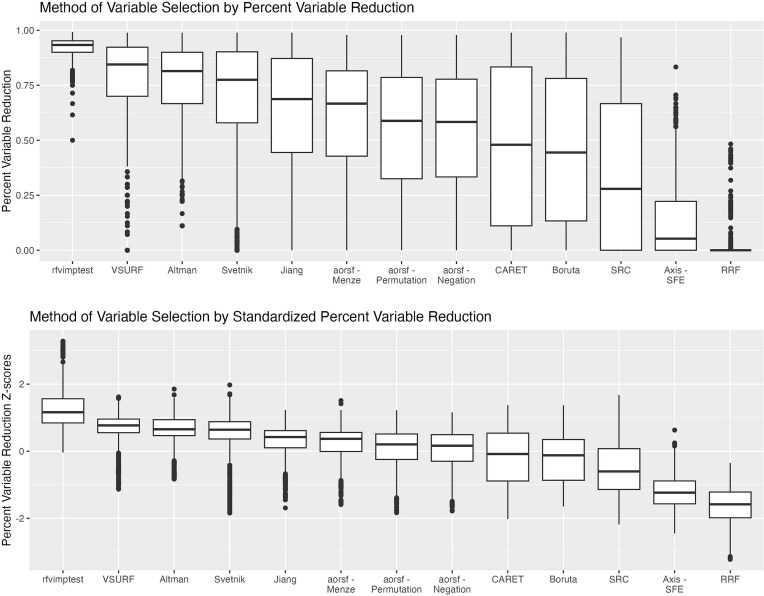
Distributions of percent reduction (absolute and standardized). This shows the distribution of percent variable reduction and standardized percent variable reduction (*z*-scores) across all replication (20 replications per dataset) over each of the 59 datasets by method of variable selection. Percent variable reduction is calculated as the percent reduction in the number of variables in each data set, relative to number of variables in the full dataset. The figure is ordered from left to right by median value in terms of highest percent reduction (fewest variables retained) to lowest percent reduction (most variables retained).

**Figure 4:**
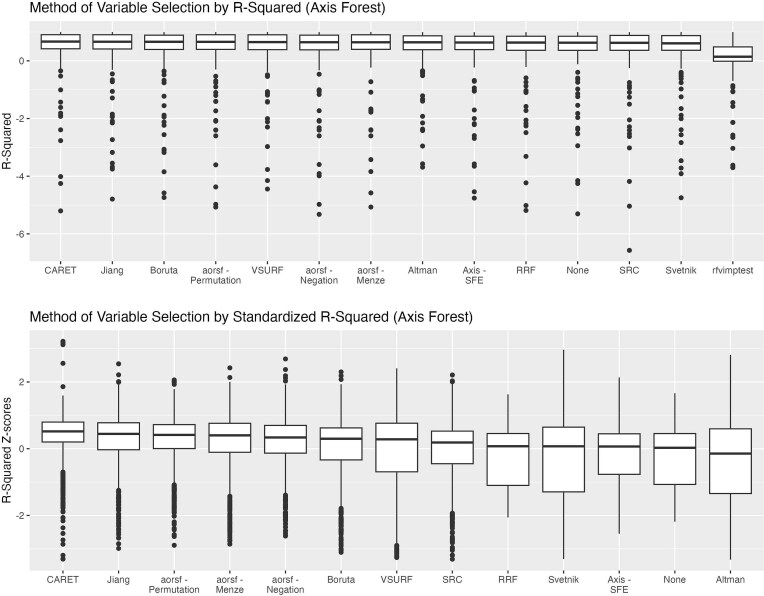
*R* ^2^ for axis forests variable selection method. For downstream axis forests, this figure shows the distribution of *R*^2^ and standardized *R*^2^ (*z*-scores) across all replication (20 replications per dataset) over each of the 59 datasets by method of variable selection. Rfvimptest is omitted from the calculation of the standardized distribution to allow a relative comparison among the more similarly performing methods. The figure is ordered from left to right by median value in terms of most accurate (highest median *R*^2^) to least accurate (lowest median *R*^2^).

**Figure 5:**
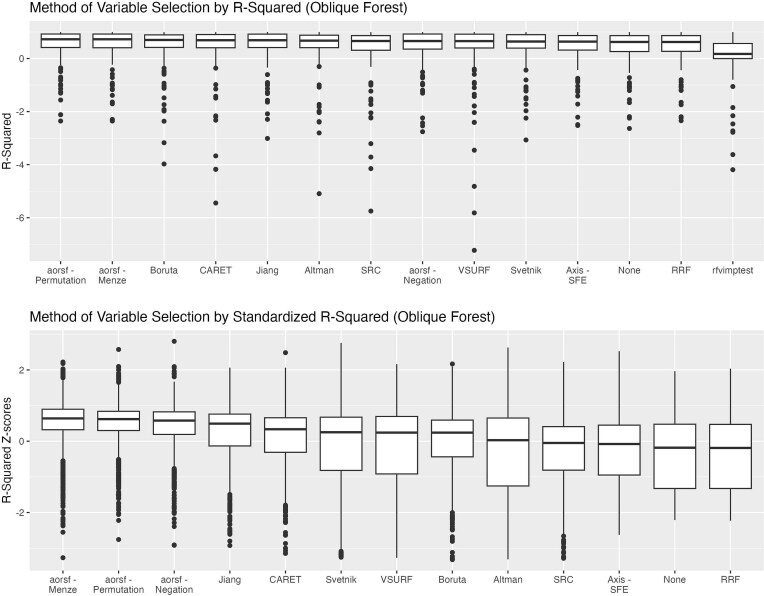
*R* ^2^ for oblique forests variable selection method. For downstream oblique forests, this figure shows the distribution of *R*^2^ and standardized *R*^2^ (*z*-scores) across all replication (20 replications per dataset) over each of the 59 datasets by method of variable selection. Rfvimptest is omitted from the calculation of the standardized distribution to allow a relative comparison among the more similarly performing methods. The figure is ordered from left to right by median value in terms of most accurate (highest median *R*^2^) to least accurate (lowest median *R*^2^).

We present descriptive statistics for *R*^2^ across the 13 variable selection methods (plus RF with no variable selection) for both axis-based and oblique RFs, along with computation time (in seconds) and percent variable reduction in Table 2. Figures 2–5 demonstrate both original and standardized distributions by replicated sample for log-time, percent variable reduction. RF with no variable selection is included in Figs. 4 and 5 for accuracy comparison but excluded from the computation time and percent reduction in Figs. 2 and 3.

**Table 2:** Distribution of *R*^2^, computation time, and percent variable reduction for variable selection procedures

	*R* ^2^ (Axis)	*R* ^2^ (Oblique)	Variable percent reduced	Time (s)
Altman				
Mean (SD)	0.55 (0.013)	0.57 (0.013)	0.74 (0.006)	299.49 (16.903)
Median [IQR]	0.64 [0.385, 0.868]	0.67 [0.409, 0.88]	0.81 [0.667, 0.9]	46.04 [16.797, 332.425]
aorsf-Menze				
Mean (SD)	0.56 (0.014)	0.6 (0.012)	0.61 (0.008)	10.68 (0.972)
Median [IQR]	0.64 [0.399, 0.904]	0.73 [0.407, 0.924]	0.67 [0.428, 0.816]	1.41 [0.429, 5.248]
aorsf-Permutation				
Mean (SD)	0.56 (0.015)	0.6 (0.012)	0.55 (0.008)	33.51 (3.786)
Median [IQR]	0.66 [0.398, 0.905]	0.73 [0.416, 0.924]	0.59 [0.325, 0.786]	2.38 [0.616, 8.964]
Boruta				
Mean (SD)	0.55 (0.015)	0.58 (0.013)	0.46 (0.009)	28.57 (2.265)
Median [IQR]	0.66 [0.396, 0.891]	0.7 [0.419, 0.893]	0.44 [0.133, 0.781]	2.91 [1.42, 15.103]
CARET				
Mean (SD)	0.58 (0.014)	0.58 (0.014)	0.47 (0.01)	3223.25 (249.952)
Median [IQR]	0.67 [0.419, 0.907]	0.69 [0.407, 0.905]	0.48 [0.111, 0.833]	176.54 [48.188, 1307.271]
rfvimptest				
Mean (SD)	0.22 (0.014)	0.27 (0.013)	0.92 (0.001)	1561.53 (130.588)
Median [IQR]	0.14 [−0.013, 0.484]	0.17 [−0.001, 0.568]	0.93 [0.9, 0.952]	46.53 [11.696, 560.366]
Jiang				
Mean (SD)	0.57 (0.014)	0.59 (0.012)	0.64 (0.008)	486.09 (37.672)
Median [IQR]	0.66 [0.411, 0.906]	0.69 [0.415, 0.921]	0.69 [0.444, 0.872]	33.14 [8.751, 193.76]
SRC				
Mean (SD)	0.55 (0.015)	0.55 (0.014)	0.36 (0.009)	26.27 (0.587)
Median [IQR]	0.63 [0.365, 0.882]	0.66 [0.312, 0.865]	0.28 [0, 0.667]	17.64 [11.65, 40.215]
aorsf-Negation				
Mean (SD)	0.55 (0.015)	0.58 (0.012)	0.54 (0.008)	26.94 (2.691)
Median [IQR]	0.65 [0.382, 0.892]	0.66 [0.356, 0.924]	0.58 [0.333, 0.778]	1.87 [0.539, 9.178]
None				
Mean (SD)	0.54 (0.014)	0.55 (0.011)		
Median [IQR]	0.63 [0.365, 0.853]	0.63 [0.263, 0.864]		
Axis-SFE				
Mean (SD)	0.55 (0.014)	0.56 (0.011)	0.13 (0.005)	0.28 (0.023)
Median [IQR]	0.64 [0.39, 0.854]	0.64 [0.319, 0.867]	0.05 [0, 0.222]	0.05 [0.022, 0.174]
RRF				
Mean (SD)	0.54 (0.014)	0.55 (0.011)	0.01 (0.002)	1.72 (0.093)
Median [IQR]	0.63 [0.366, 0.855]	0.62 [0.271, 0.864]	0 [0, 0]	0.32 [0.112, 1.548]
Svetnik				
Mean (SD)	0.55 (0.014)	0.58 (0.012)	0.69 (0.008)	1211.82 (70.736)
Median [IQR]	0.61 [0.366, 0.875]	0.64 [0.393, 0.901]	0.78 [0.579, 0.902]	151.56 [58.179, 982.864]
VSURF				
Mean (SD)	0.56 (0.014)	0.57 (0.016)	0.76 (0.007)	245.03 (20.316)
Median [IQR]	0.65 [0.388, 0.903]	0.66 [0.398, 0.919]	0.84 [0.7, 0.923]	20.12 [8.302, 107.175]

In terms of computation time (Fig. 2), the fastest variable selection methods were Axis-SFE, RRF, aorsf-Menze, aorsf-Negation, and aorsf-Permutation, each faster than average (i.e. *z*-scores <0 on the standardized plot), with median computation times <5 s. The least efficient methods were rfvimptest*,* CARET, and Svetnik. Generally, each method demonstrated similar variability in computation time across dataset replications, except for SRC, which had less variability compared to other methods.

Percent variable reduction (Fig. 3) ranged from a median of >90% for rfvimptest to near 0% for RRF. The most restrictive methods achieving the largest median percent reduction were rfvimptest (93%), *VSURF* (84%), Altman (81%), and Svetnik (78%). The selection methods *CARET*, Boruta, and SRC had the greatest variability in percent variable reduction, demonstrating the widest interquartile ranges (IQRs) for both absolute percent reduction as well as standardized percent reduction. In contrast*,* rfvimptest and RRF had the smallest variability in percent reduction.

**Figure 6:**
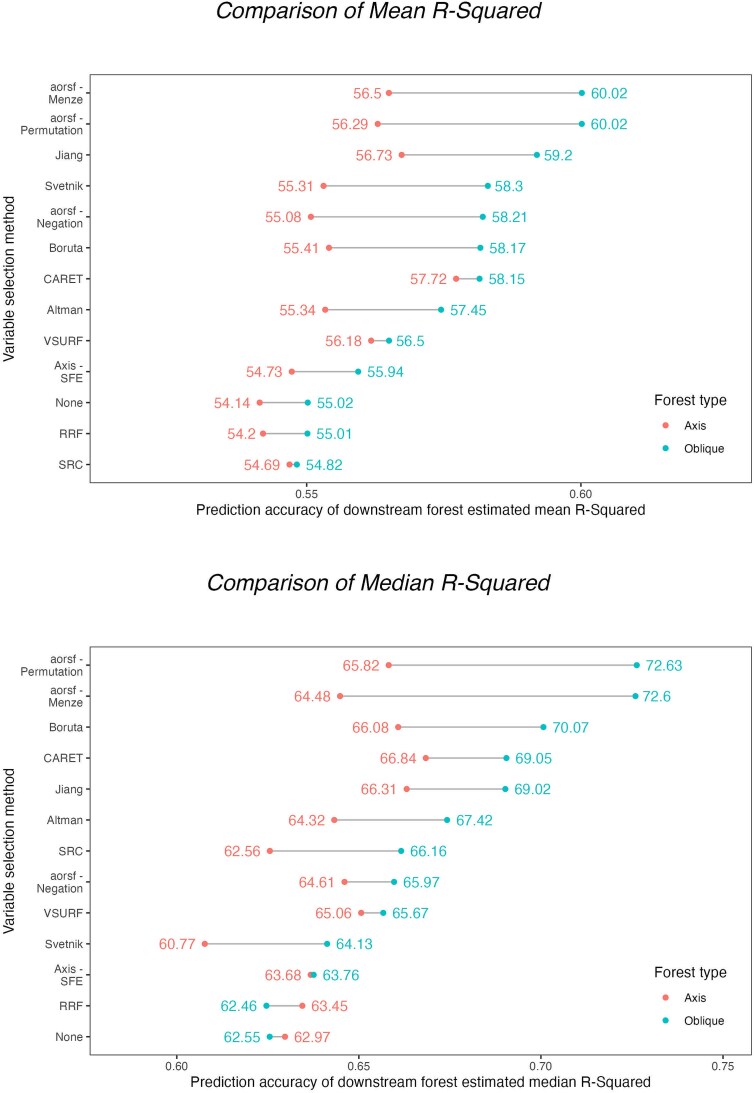
Comparison of mean and median *R*^2^ by forest type and method of variable selection. A comparison of median *R*^2^ across between downstream axis and oblique forests across all dataset replications by method of variable selection.

The distribution of median *R*^2^ across datasets by method of selection for axis-based (Fig. 4) or oblique (Fig. 5) RF models ranged from 0.61 to 0.67 and 0.62 to 0.73, respectively, for all variable selection methods, with the exception of rfvimptest which achieved a median *R*^2^ across datasets of 0.14 and 0.17 for axis-based and oblique RFs, respectively. With the exclusion of rfvimptest, the standardized distribution demonstrates that all methods are within ±1 SD of average performance by dataset replication. Comparing ranges in performance within methods across dataset replications, VSURF, Svetnik, RRF, and Altman had slightly larger IQR spreads compared to other methods for both axis-based and oblique forests based on standardized comparisons (Figs. 4 and 5). The mean *R*^2^ of downstream oblique RFs was higher than axis-based RFs for all variable selection methods (Fig. 6; top panel). Similarly, oblique RFs obtained higher median *R*^2^ for all variable selection methods except for RRF and no variable selection (Fig. 6; bottom panel). Overall, the highest mean and median *R*^2^ were the oblique RFs using aorsf-Menze and the aorsf-Permutation method for variable selection.

**Figure 7:**
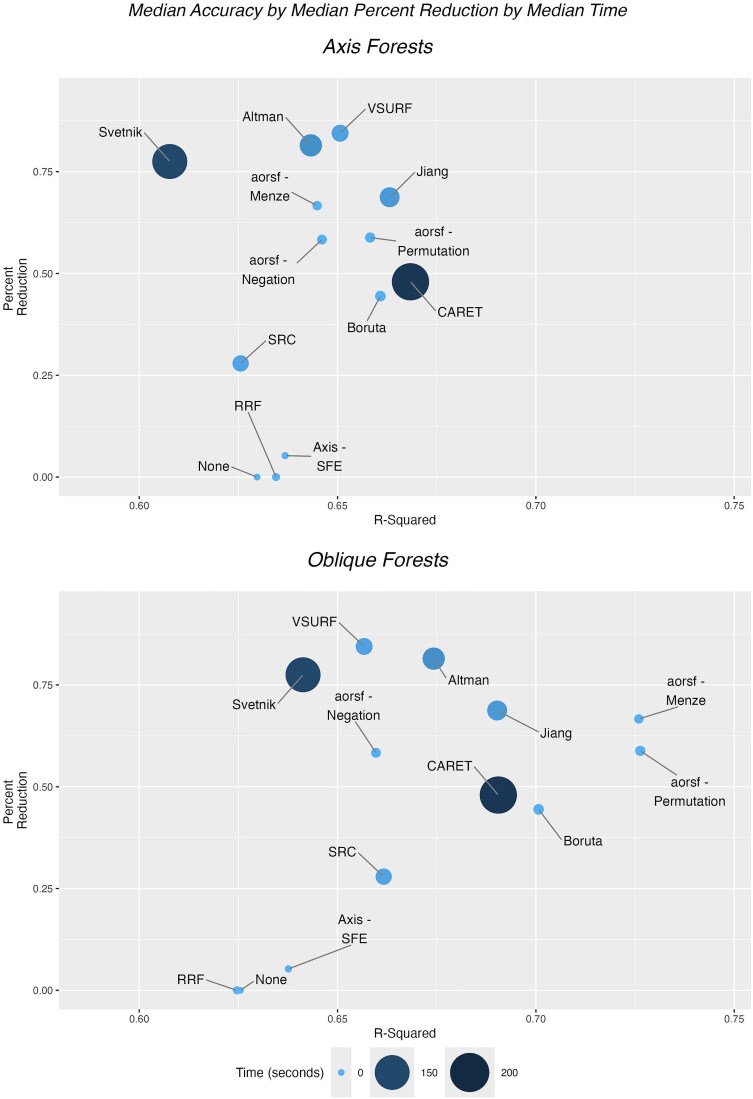
Comparison of accuracy by time and percent reduction. A comparison of variable selection methods by accuracy as measured by *R*^2^, percent reduction in variables, and computation time. The top-performing methods are those farthest to the right in the plot, and larger and darker bubbles represent greater computation time.

Finally, we compare performance based on all three metrics considered jointly: median accuracy measured by *R*^2^, median percent reduction in variables, and median computation time (Fig. 7). The variable selection methods that produced downstream prediction models with the highest accuracy were CARET, Boruta, aorsf-Permutation, and Jiang for axis-based RFs and aorsf-Permutation and aorsf-Menze for oblique RFs. The most parsimonious models with the highest median percent reduction in variables were VSURF and Altman, with median percent reduction of >80%, while Svetnik, aorsf-Menze, and Jiang were the next most parsimonious with median percent reduction >65%. The fastest methods were aorsf-Permutation, Axis-SFE, RRF, aorsf-Negation, Boruta, and aorsf-Menze. Considering all three metrics together for axis-based RFs, Boruta and the three aorsf methods demonstrated high *R*^2^, low computation time, and high percent reduction in variables relative to the other methods (Fig. 7). Jiang’s method, CARET, and VSURF had high *R*^2^ but also had higher computation time. For oblique RFs (Fig. 7), aorsf-Permutation and aorsf-Menze result in high *R*^2^, low computation time, and high percent reduction in variables relative to the other methods.

### Sensitivity analysis: excluding runs for which any method had no variables selected

Conducting this benchmarking study, we found that some methods did not select any variables for certain datasets and splits of the data (i.e. simulation runs). We summarize the number of times this occurred, across all replications, and the number of times it occurred in at least one replication by dataset (Table 3). Notably, in 380 out of 1180 replications (59 datasets by 20 replications each), and occurring in 47 of the 59 datasets, no variables were selected based on the method in the R package rfvimptest. This issue also occurred in Boruta (2 datasets), Altman’s method (9 datasets), and VSURF (16 datasets). The other methods not included in Table 3 selected variables for every simulation run.

**Table 3:** Instances when no variables were selected

Method	No. of times occurred	No. of datasets occurred in
Altman	55	9
Boruta	4	2
rfvimptest	380	47
VSURF	86	16

**Figure 8:**
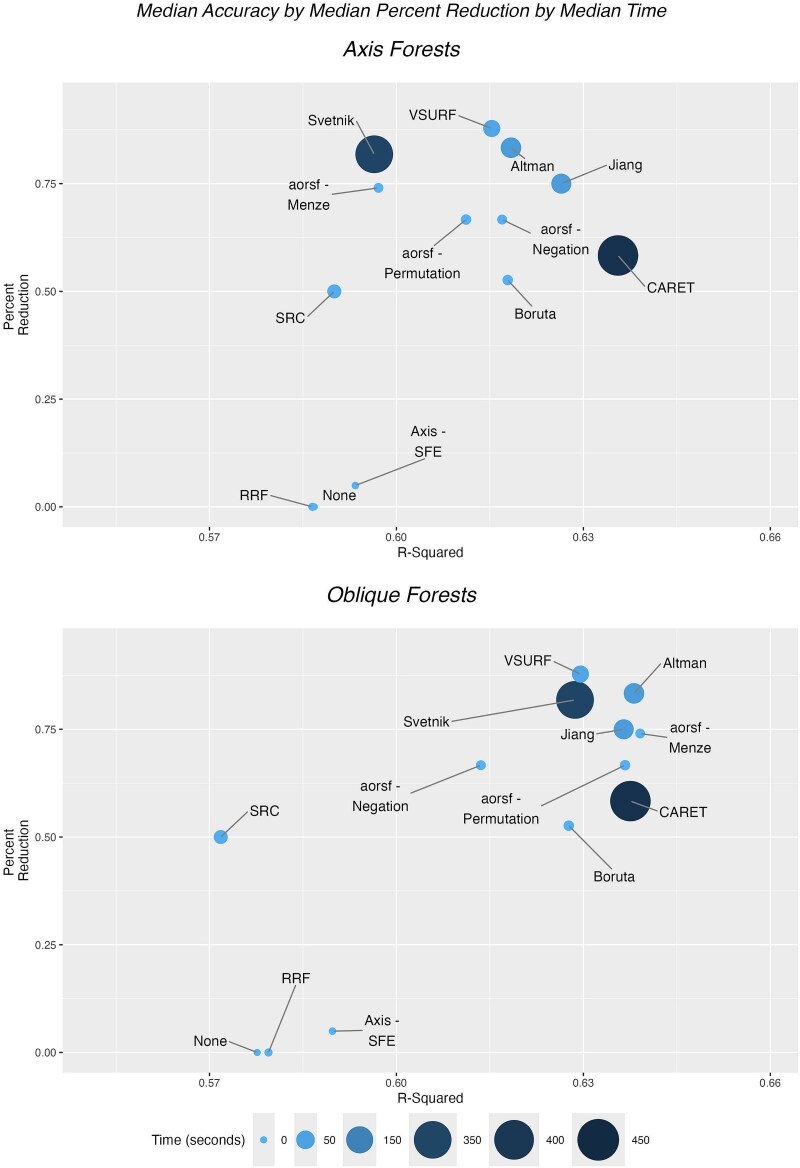
Comparison of accuracy by time by percent reduction (complete case). Among only replications in which all methods identified at least one variable, a comparison of variable selection methods by accuracy as measured by *R*^2^, percent reduction in variables, and computation time. The top-performing methods are those farthest to the right in the plot, and larger and darker bubbles represent greater computation time.

As a sensitivity analysis, we present the comparison of accuracy by percent reduction and time for a complete case analysis, for which we compare methods only for dataset replications where all methods selected at least one variable (Fig. 8). This sensitivity analysis consisted of 10 192 replications (compared to 16 520 in the primary analysis) across 58 datasets. The median performance in terms of *R*^2^ decreased across all datasets with the elimination of the datasets completely excluded; however, the ranking of variable selection methods by performance remains similar to the primary analysis. For axis-based RFs, Boruta, aorsf-Permutation, and aorsf-Negation had high *R*^2^, low computation time, and high percent reduction in variables selected. When ignoring computation time, methods that had high *R*^2^ include CARET, Jiang, Altman, and VSURF. For oblique RFs, aorsf-Menze and aorsf-Permutation had high *R*^2^, low computation time, and high percent reduction in variables selected. Without considering computation time, the methods with highest *R*^2^ are Altman, Jiang, and CARET, as well as aorsf-Menze and aorsf-Permutation.

### Results for datasets with high versus low N:P ratio

**Figure 9:**
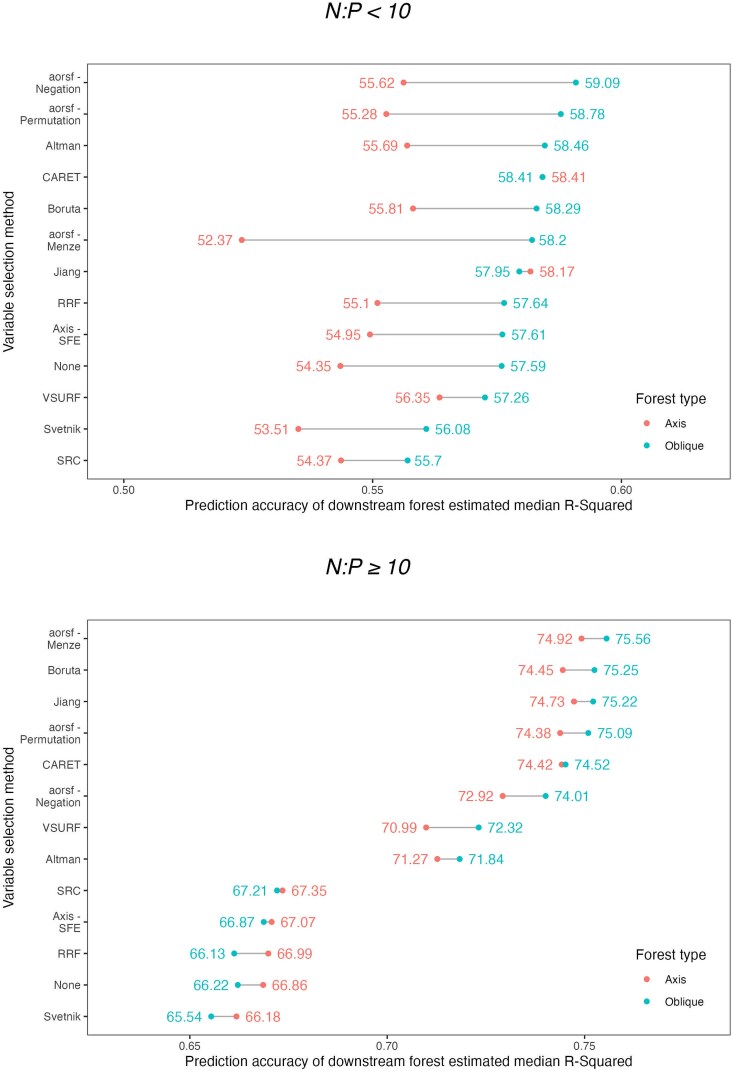
Median *R*^2^ by variable selection type based on low versus high N:P. A comparison of accuracy between downstream oblique versus axis random forest by variable selection method, stratified by high (≥10) versus low (<10) N:P ratios. High N:P ratio represents dataset in which there were ≥10 observations for each included predictor.

We conducted a subgroup analysis by datasets that had differing ratios of the sample size to the number of predictors (Fig. 9). There were 13 datasets with a low ratio (N:P < 10) and the remaining datasets had a high ratio (N:P ≥ 10). In the scenario of low N:P ratio, the difference between *R*^2^ values for oblique and axis-forests was larger than in high N:P datasets, with oblique forests providing a better fit than axis forests with a median *R*^2^ difference >0.02 among the top-performing variable selection methods (aorsf-Negation, aorsf-Permutation, and Altman). However, for datasets with high N:P, the downstream results are closer between forests types, with the difference in median *R*^2^ between RFs among the top-performing variable selection types being <0.01. In the scenario with high N:P, methods that had the highest *R*^2^ were aorsf-Menze, Boruta, Jiang, aorsf-Permutation, and CARET.

### Comparing methods grouped by characteristics of the methods

#### Results for comparing axis-based, conditional, and oblique RFs in the variable selection method

The type of RF implemented in each of the variable selection methods differed (Table 1). Most methods used standard RF, with some using conditional RF (Svetnik’s method and Jiang’s method) and some using oblique RF (aorsf-Menze, aorsf-Negation, and aorsf-Permutation). In this section, we analyze Fig. 7 by RF implementation type. Methods employing conditional RF were computationally slow. The methods using oblique RF performed had low computation time and high accuracy, although aorsf-Negation had lower accuracy when oblique RF was also used in the modeling. Of the methods using standard RF, Boruta had high accuracy combined with low computation time, while CARET had fairly high accuracy it had longer median computation times.

#### Results for comparing test-based and performance-based methods

In addition to the type of RF method used in the variable selection, we categorize methods as test based and performance based (Table 1). Test-based methods use tests on the variables, either statistical or permutation based, to determine which variables should be in the model, and included Altman, Boruta, aorsf-Permutation, Axis-SFE, and rfvimptest. The other methods were all considered performance based, which use changes in model performance to determine variables to include. Comparing performance via accuracy, percent variable reduction, and time in Fig. 7, there were no discernable patterns when comparing test-based and performance-based methods.

## Discussion

We presented a comparison of R implementations of variable selection methods for RF modeling of continuous outcomes using 59 datasets. Variable selection methods assessed were based on axis-based, conditional, and oblique RFs. The best methods were those that provide the best accuracy in terms of *R*^2^ first and foremost, and parsimony favored among competing methods with comparable accuracy. Of secondary interest was computation time.

Across all metrics considered, a top-performing variable selection method was aorsf-Permutation, an approach based on oblique RFs. This method was among the best performers in terms of *R*^2^, with a median computation time <3 s and a median of 59% variable reduction across the random dataset split replications. Aorsf-Permutation method provides good results for downstream fitted models using oblique RFs and performs well for data with both low and high N:P ratios. Boruta, CARET, and Jiang’s method performed slightly better than aorsf-Permutation for axis-based RF, but had a lower median *R*^2^ than aorsf-Permutation for oblique RF. aorsf-Menze performed similarly to aorsf-Permutation for oblique RFs, and slightly worse than aorsf-Permutation in axis-based RFs. RRF and axis-SFE perform similarly in computation time to aorsf-Permutation, Boruta, and aorsf-Menze, but have slightly worse performance in terms of *R*^2^ and variable percent reduction. CARET had good accuracy in terms of *R*^2^ whereas Svetnik had poorer accuracy with both methods achieving high computation times. Rfvimptest had the highest computation time and largest percent reduction in variables and failed to select a single variable in a large proportion of the simulation runs. Analyzing the extremes from the metric percent reduction in variables across the datasets and replications, RRF retained the most variables, whereas rfvimptest retained the least variables.

A novel contribution of our study is consideration of axis-based and oblique RF implementations. We found that oblique fitted RFs generally led to better overall accuracy in terms of *R*^2^ than axis-based RFs, regardless of the methodology used for variable selection. In the overall results, the magnitude of this difference was largely driven by datasets with a low N:P ratio. In our secondary analyses, we found that in datasets with <10 observations per included predictor, oblique RFs provided better accuracy than axis-based RFs, which is consistent with previous investigations of oblique RFs [24]. For datasets with a N:P ratio >10, the differences between axis-based and oblique RFs for a given variable selection method were much smaller (within 1% in *R*^2^) for the top 5 performing variable selection methods.

Based on our benchmarking design, we were able to compare performance characteristics of different RF variable selection methods implemented in R. The differences that we observed may be explained by general mechanics in the variable selection methods (e.g. recursive feature elimination techniques tended to provide the highest downstream prediction accuracy) and could also be explained by characteristics of datasets (e.g. oblique RF variable selection methods did exceptionally well in data with a high number of predictors compared to observations).

Our results should be considered in the greater context of previous literature comparing RF variable selection methods. Most previous work has focused on classification and prediction of categorical outcomes [7–10, 31]. These studies suggested that VSURF [20] and Boruta [17] may be preferable. In our previous work for categorical outcomes [11], VSURF [20] and Jiang’s method [22] were identified as optimal for datasets with binary outcomes, whereas varSelRF [27] and Boruta [17] were preferable for large datasets. Degenhardt [8] used simulated and real data for continuous outcomes and found that Boruta [17] and Altmann’s method [32] are suitable for low dimensional data. This is consistent with our finding that Boruta [17] is a top performer; however, the study by Degenhardt and colleagues [8] did not include oblique RF methods or implementations as in our study. VSURF [20] did not perform as well as other methods in our study for continuous outcomes, which differs from previous work that identified it as a top performer for classification models [11]. Another major difference between our work and previous studies is that our implementations of methods for variable selection using conditional inference were not optimal performers. This is largely because conditional inference RFs have high computation times, whereas axis-based and oblique RF implementations have implementations that are quite fast.

There are some limitations of our study. We were constrained to 59 datasets that were freely available in R packages. Regression datasets with continuous outcomes are less available than classification datasets as analyzed in our previous work [11]. However, this is a sizeable number of datasets from different domains, and it provides a suitable basis for benchmarking RF variable selection methods. A few of the datasets had missing values, but we decided to include them to increase the number of datasets used in the benchmarking study. Given that only 7 of the 59 datasets had missing values, the imputation of missing values likely did not bias the study results. A future study could investigate the impact of missing data on RF variable selection for regression modeling. Our study focused on RF methods, and this may limit the generalizability of findings since variable selection may vary across different classifiers. Future studies could replicate our analysis using different classifiers. We also only included variable selection methods specific to RF; additional work could investigate general variable selection methods that are not RF specific such as use of Shapley Additive exPlanation values or statistical metrics such as F1. A limitation of our study is that we did not conduct hyperparameter tuning for the models and elected to use default settings that may not provide optimal performance. Default hyperparameter values are often arbitrary and therefore may distort the comparison of methods. Despite these limitations, our study provides a thorough benchmarking experiment to compare implementations of RF variable selection methods in terms of accuracy, computational efficiency, and parsimony.

A primary contribution of our study is the ability to assess RF variable selection methods for continuous outcomes using axis-based and oblique RF models with default implementations in R. Based on our benchmarking study of 59 datasets and optimizing accuracy, computational efficiency, and parsimony, Boruta and aorsf-Permutation may be preferable for axis-based RF models, whereas aorsf-Permutation and aorsf-Menze may be preferable for oblique RF models. For applied analyses, we recommend researchers try several of the top-performing methods and compare performance in order to identify the best method for their specific dataset and application.

**Key Points**

- There are many variable selection methods available for developing random forest models for continuous outcomes and little guidance for choosing which method may be best for different types of datasets.
- Using a benchmarking approach, we compare prediction accuracy (*R*^2^), parsimony (percent reduction in the number of variables included), and computation time for available variable selection methods for random forest for continuous outcomes in 59 freely available datasets using R software.
- Variable selection methods in the R packages *Boruta* and *aorsf* may be preferable for continuous outcome modeling with random forest since they had the highest *R*^2^ and percent reduction in variables and lowest computation time.

## Supplementary Material

RVFS_Supplemental_File_1_bbaf096

## Data Availability

The data and code to generate results for this article are available on GitHub and can be accessed at https://github.com/NateOConnellPhD/rfvs_regression.
